# Aberrant Hippo-YAP/TEAD Signaling Drives Malignant Transcriptional Reprogramming in External Auditory Canal Squamous Cell Carcinoma

**DOI:** 10.1158/2767-9764.CRC-25-0626

**Published:** 2026-02-02

**Authors:** Kuniaki Sato, Noritaka Komune, Mayumi Ono, Shinsaku Itoyama, Takahiro Hongo, Takafumi Nakano, Kensuke Koike, Kenichi Taguchi, Koshi Mimori, J. Silvio Gutkind, Muneyuki Masuda, Takashi Nakagawa

**Affiliations:** 1 Moores Cancer Center, University of California San Diego, La Jolla, California.; 2 Department of Otorhinolaryngology, Graduate School of Medical Sciences, Kyushu University, Fukuoka, Japan.; 3 Department of Surgery, Kyushu University Beppu Hospital, Oita, Japan.; 4 Department of Head and Neck Surgery, National Hospital Organization Kyushu Cancer Center, Fukuoka, Japan.; 5 Department of Pathology, National Hospital Organization Kyushu Cancer Center, Fukuoka, Japan.

## Abstract

External auditory canal squamous cell carcinoma (EACSCC) is an exceptionally rare malignancy related to chronic tissue damage and inflammation. The molecular underpinnings of EACSCC are poorly understood, and evidence-based therapeutic strategies are not fully developed. In this study, we performed integrated multiomics analyses of RNA sequencing (RNA-seq) and chromatin immunoprecipitation sequencing (ChIP-seq) for Yes-associated protein (YAP) and histone 3 lysine 27 acetylation (H3K27Ac) in primary EACSCC and noncancerous ear skin samples. RNA-seq indicated hyperactivation of the YAP/TEA domain family members (TEAD)–mediated transcriptional program in EACSCC, which was significantly correlated with poor clinical outcomes. ChIP-seq suggested gained accessibility for transcription factor (TF) binding sites for TEAD, AP-1, and paired-like homeodomain (PITX) TFs in EACSCC and the presence of EACSCC-specific super-enhancers (SE). Importantly, YAP-bound SEs were involved in oncogenic transcription, including EGFR signaling. For further validations, functional experiments were performed *in vitro* and *in vivo*. The small-molecule TEAD inhibitor (smTEADi) VT104 significantly suppressed the proliferation and clonogenicity of EACSCC-derived cells. Interestingly, smTEADi not only inhibited the YAP–TEAD interaction but also induced YAP–PITX2 binding, suggesting that PITX2 could represent an alternative partner TF of YAP under TEAD-inhibited conditions in EACSCC. Knockdown of PITX2 enhanced sensitivity to VT104, inhibiting cell growth and migration of EACSCC and head and neck squamous cell carcinoma cells, whereas overexpression of PITX2 induced oncogenic gene expression programs, as well as YAP/TEAD target genes, promoting tumor growth *in vivo*. Of note, nuclear YAP and PITX2 were coexpressed in primary EACSCC tissues and significantly correlated with the poor prognosis of patients with EACSCC. Together, this study highlighted the hyperactivated YAP-driven transcriptional program and its potential as a therapeutic target in EACSCC.

**Significance:**

This study provides evidence for the hyperactivation of YAP/TEAD-driven transcriptional programs in EACSCC, an exceptionally rare malignancy related to chronic tissue damage and inflammation. A comprehensive multiomics approach, including YAP and H3K27Ac ChIP-seq in clinical samples, not only suggested hyperactivation of YAP/TEAD but also identified YAP–PITX2 as a potential oncogenic transcriptional machinery under TEAD-inhibited conditions. Our results may provide a better understanding of EACSCC and contribute to the future development of therapeutic strategies.

## Introduction

The notion “*Tumors are wounds that do not heal*” suggests the close links among cancer, inflammation, tissue damage, and wound healing (1). The similarity between cancer onset/progression and the wound healing process has long been described, in which signaling pathways and microenvironmental changes are shared in many aspects (2–4). Importantly, recent studies revealed that nongenetic alterations, or “transcriptional reprogramming,” including chromatin remodeling and aberrant transcription, may regulate these processes (5, 6). One of the most important molecules for this reprogramming is Yes-associated protein (YAP), a transcriptional coactivator negatively regulated by the Hippo pathway (7). Mechanistically, YAP drives the transcription of target genes cooperatively with partner transcription factors (TF), especially with TEA domain family members (TEAD), in response to mechanical and microenvironmental stress (8, 9). Indeed, several lines of evidence have shown crucial roles of YAP in wound healing and tissue regeneration (10–12) as well as carcinogenesis (13–15), suggesting that YAP acts as a transcriptional driver of malignant transformation and tumor progression in multiple tumor types.

External auditory canal squamous cell carcinoma (EACSCC) is an exceptionally rare malignancy, reportedly diagnosed in one to six out of a million individuals annually, arising from the squamous epithelium of the external auditory canal (16). The biological and molecular underpinnings of EACSCC, as well as evidence-based therapeutic strategies for this malignancy, have not yet been fully established due to its rarity. Unlike head and neck squamous cell carcinoma (HNSCC) or skin basal cell carcinoma, smoking or UV exposure is not evident in the mutational signature of EACSCC, as we previously reported by whole-exome sequencing (17), and the presence of high-risk human papillomavirus (HPV) infection is extremely rare (18). Clinically, risk factors of EACSCC include chronic tissue damage and inflammation induced by mechanical stimuli (e.g., habitual ear picking, especially in Eastern Asian countries; ref. 19), which may suggest that continuous and aberrant hyperactivation of YAP-driven regenerative transcriptional programs could drive the malignant initiation and progression of EACSCC. Moreover, we previously showed that mutations recurrently observed in EACSCC included loss-of-function (LOF) mutations of FAT atypical cadherin 1 (*FAT1*), which is an upstream regulator of the Hippo pathway (17). LOF of FAT1 results in Hippo pathway dysfunction and subsequent YAP hyperactivation, as we previously reported in HNSCC (20). Taken together, we hypothesized that YAP-driven transcriptional programs could be a crucial oncogenic driver of EACSCC.

Here, we report the transcriptomic and epigenetic aberrations of EACSCC. Comprehensive analysis of RNA sequencing (RNA-seq) and chromatin immunoprecipitation sequencing (ChIP-seq) in clinical EACSCC samples and noncancerous ear skin tissues uncovered hyperactivation of YAP and downstream pathway aberrations. Additionally, we revealed a potential alternative transcriptional machinery of YAP under TEAD-inhibited conditions, and utilizing multiple experimental strategies, including a small-molecule TEAD inhibitor (smTEADi) and EACSCC-derived cells, we investigated the clinical significance of YAP hyperactivation in EACSCC.

## Materials and Methods

### Ethics statement

The protocol of this study was reviewed and approved by the Institutional Review Boards and ethics committees of Kyushu University (protocol numbers: 700-2 and 30-268). All experiments with human samples were conducted according to the principles expressed in the Declaration of Helsinki.

### Patients and sample collection

All patients diagnosed with EACSCC and treated at Kyushu University Hospital Department of Otolaryngology from September 2015 to March 2019 provided written informed consent and were enrolled in this study. Primary tumor samples were collected by tissue biopsy or surgical resection. Noncancerous ear skin tissues were collected from surgically resected tissues.

### RNA extraction, reverse transcription, and qPCR

Total RNA was isolated from EACSCC and noncancerous ear skin tissues using a NucleoSpin RNA Plus kit (Macherey-Nagel). Reverse transcription was performed using a PrimeScript RT Master Mix (Takara Bio), followed by qPCR in a CFX96 Real-Time PCR Detection System (Bio-Rad Laboratories, RRID:SCR_018064) with TB Green Premix Ex Taq II (Tli RNase H Plus; Takara Bio). The primers used in this study are listed in Supplementary Table S2. β-Actin was used as an internal control. The mRNA expression levels were calculated using the 2^−ΔΔCt^ method.

### RNA-seq and analysis

RNA extracted from EACSCC and skin tissues was sequenced on a DNBSEQ-G400 sequencer (RRID:SCR_017980) at Beijing Genomics Institute (RRID:SCR_011114). The sequencing reads were aligned to the human reference GRCh38/hg38 genome by STAR version 2.7.9 (RRID:SCR_004463) using GENCODE version 38 annotations. Gene count tables were generated using RNA-Seq by Expectation Maximization (RRID:SCR_000262). Downstream analyses were carried out using R version 4.4.1 (R Project for Statistical Computing, RRID:SCR_001905). Normalization of the read count data and the detection of differentially expressed genes (DEG) between EACSCC and skin were carried out with DESeq2 version 1.10.1 (RRID:SCR_015687). For sample clustering and principal component analysis (PCA), genes with zero counts across all samples were removed from the analysis. Gene Ontology (GO) analysis of DEGs was performed using Molecular Signature Database Hallmark pathways, GO biological process, and Kyoto Encyclopedia of Genes and Genomes pathways in the R package enrichR (RRID:SCR_001575). Gene set enrichment analysis (GSEA) was performed using GSEA version 4.3.2 (RRID:SCR_003199; ref. 21). Single-sample GSEA (ssGSEA) was performed in GenePattern (RRID: SCR_003201). TF activity prediction was performed using the R package decoupleR version 2.9.1 (RRID:SCR_027127; ref. 22). YAP/TAZ-TEAD transcriptional target signature gene sets were obtained from previous studies (23, 24).

### ChIP-seq and analysis

Frozen tumor samples and skin tissue samples were sent to Active Motif (RRID:SCR_013589) for ChIP reaction, library preparation, and sequencing. Cross-linked cells were solubilized using a lysis protocol, sonicated, and immunoprecipitated with antibodies for histone 3 lysine 27 acetylation (H3K27Ac; #39133, Active Motif, RRID:AB_2561016) and YAP (#Y1200-01D, US Biological, RRID:AB_2927438). Immunoprecipitated chromatin samples were sequenced on an Illumina NextSeq 500 sequencer (RRID:SCR_014983). The 75 bp single-end raw reads were aligned to the human reference genome GRCh38/hg38 using BWA version 0.7.12 (RRID:SCR_010910). Only reads aligned with no more than two mismatches and mapped uniquely to the genome were used in the subsequent analysis. PCR-duplicated reads were removed. Significant peaks were identified using MACS2 version 2.1.0 (RRID:SCR_013291) with a *P* value = 1 × 10^−7^ cutoff, and respective input samples were used as background. For visualization of the called peaks and regions, deepTools (RRID:SCR_016366) and pyGenomeTracks (RRID:SCR_025312) were used. ROSE (RRID:SCR_017390) was applied for the detection of super-enhancers (SE) using default parameters (25). Calculation of overlaps among SE regions was performed using the *multiinter* function of BEDTools (RRID:SCR_006646), and the regions with lengths less than 3 kb were filtered out. SE regions observed in at least two EACSCC samples, but never observed in noncancerous skin samples, were defined as EACSCC-specific SEs. Enhancer–gene interactions in the detected enhancers and SEs were estimated using GeneHancer version 5.24 (RRID:SCR_023953), a comprehensive enhancer/promoter database of the human genome (26). The association between enhancers and transcriptional target genes in this database was estimated using a combination of multiple methods, including distance-based associations (nearest gene neighbors and proximity to transcription start sites), capture Hi-C promoter–enhancer interactions, and enhancer RNA expression in human cells and tissues, as previously described (26). The overlap between the significant H3K27Ac peak regions that compose SEs and the annotated enhancers included in GeneHancer was calculated for the prediction of the transcriptional target genes of SEs. Regions annotated as promoters were removed. Nucleosome-free regions (NFR) in H3K27Ac peak regions were estimated using the HisTrader algorithm (27). Detected YAP peaks were annotated to putative transcriptional target genes using GREAT (RRID:SCR_005807; ref. 28) with the *basal-plus extension* option (2 kb upstream and 2 kb downstream, distal up to 1,000 kb options). TF binding motif discovery analysis in 200 bp regions around YAP peak summits and motif enrichment analysis in NFRs were performed using HOMER version 5.1 (RRID:SCR_010881; ref. 29) with the default database included in HOMER and the HOCOMOCO version 13 database (RRID:SCR_005409; ref. 30).

### Cell lines and culture

Human HNSCC cell lines SCC9 (RRID:CVCL_7793) and FaDu (RRID:CVCL_1218) were obtained from the ATCC, and HSC4 (RRID:CVCL_1289) was obtained from the Japanese Collection of Research Bioresources. SCEAC-ono2 (ONO2; RRID:CVCL_D3YU) was established from a primary EACSCC (31, 32). Short tandem repeat analysis was performed using genomic DNA extracted from cell lines to exclude potential cross-contamination risks in 2023, as previously described (31). All cell lines used in this study were regularly tested for *Mycoplasma* contamination at BML, and no presence of *Mycoplasma* contamination was found, according to qPCR utilizing custom primers in an Applied Biosystems 7900HT Fast Real-Time PCR System (RRID:SCR_018060). The most recent screening was performed in December 2025. Cells were maintained in Dulbecco’s Modified Eagle Medium (DMEM)/F12 (Sigma Aldrich), supplemented with 10% fetal bovine serum (FBS; Sigma Aldrich), 0.1 mmol/L MEM nonessential amino acids solution (Thermo Fisher Scientific), 1 mmol/L sodium pyruvate (Thermo Fisher Scientific), 2 mmol/L L-glutamine (Thermo Fisher Scientific), and 1% antibiotic-antimycotic mixed solution (Nacalai Tesque) at 37°C with 5% CO_2_.

### Western blotting

Cultured cells were lysed with RIPA buffer (Thermo Fisher Scientific) containing protease inhibitor (Sigma Aldrich) on ice. Protein concentration was determined using a BCA protein assay kit (Takara Bio). A total of 30 μg protein per lane was loaded on 10% Mini-PROTEAN TGX Tris-Glycine gels (Bio-Rad Laboratories), separated by SDS-PAGE, and then transferred to polyvinylidene difluoride membranes. After blocking with 5% nonfat milk in Tris-buffered saline with 0.1% Tween 20, the membranes were incubated overnight at 4°C with the following primary antibodies: paired-like homeodomain TF 2 (PITX2; 1:500, #PA5-98817, Thermo Fisher Scientific, RRID:AB_2813430), YAP (1:1,000, #14074, Cell Signaling Technology, RRID:AB_2650491), TEAD1 (1:1,000, #12292, Cell Signaling Technology, RRID:AB_2797873), CD44 (1:1,000, #3570, Cell Signaling Technology, RRID:AB_2076465), CDH2 (1:1,000, #13116, Cell Signaling Technology, RRID:AB_2687616), Snail (1:1,000, #3879, Cell Signaling Technology, RRID:AB_2255011), and β-actin (1:1,000, #4967, Cell Signaling Technology, RRID:AB_330288). The membranes were then incubated with the goat anti-rabbit IgG/horseradish peroxidase–conjugated secondary antibody (1:2,000, #7074, Cell Signaling Technology, RRID:AB_2099233) for 1 hour at room temperature. Protein bands were detected with Clarity ECL Western Blotting Substrates and the ChemiDoc MP Imaging System (Bio-Rad Laboratories, RRID:SCR_019037).

### Coimmunoprecipitation

Coimmunoprecipitation (Co-IP) was performed using SureBeads Protein A/G magnetic beads (#161-4833, Bio-Rad Laboratories) according to the manufacturer’s protocols. Briefly, cells at 70% confluency were lysed with Pierce IP Lysis Buffer (#87787, Thermo Fisher Scientific) on ice. The total cell lysate was incubated with YAP antibody (1:100, #14074, Cell Signaling Technology, RRID:AB_2650491) or IgG for 2 hours at room temperature, followed by incubation with magnetic beads at 4°C overnight. The immunoprecipitants were washed with PBS containing 0.1% Tween 20 three times. Proteins bound to the beads were eluted using SDS sample buffer (Bio-Rad Laboratories) at 98°C for 5 minutes. Eluted samples were subjected to SDS-PAGE and Western blotting with input samples.

### Plasmid transfection and generation of stable expressing cells

pCMV6-Entry (#PS100001) control plasmid and PITX2 overexpressing plasmid (#RC204179) were purchased from OriGene. Transfection was performed using PEI MAX (#24765, Polysciences) according to the manufacturer’s instructions. Twenty-four hours after transfection, the cells were subjected to antibiotic selection in medium containing G418 (Thermo Fisher Scientific). Cells stably expressing the recombinant protein were pooled for experiments.

### siRNA-mediated knockdown experiments

Cells were seeded onto six-well plates (2 × 10^5^ cells/well) and incubated overnight. Pooled siRNAs (20 nmol/L) for PITX2 (#sc44016, Santa Cruz Biotechnology) and negative control siRNA (#sc37007, Santa Cruz Biotechnology) were transfected into cells using Lipofectamine RNAiMAX (#13778150, Thermo Fisher Scientific) according to the manufacturer’s protocol. Forty-eight hours after transfection, the cells were harvested for RNA/protein extraction. Transfected cells were used for downstream analyses.

### Cell proliferation assay

Cell viability was detected using a Cell Counting Kit-8 (CCK-8) cell proliferation assay (#CK04-05, Dojindo Molecular Technologies) according to the manufacturer’s protocol. Briefly, the cells were seeded onto 96-well plates (2 × 10^3^ cells/well) and cultured overnight at 37°C. CCK-8 solution (10 μL) was added at 24, 48, 72, and 96 hours after seeding, and the absorbance at 450 nm was measured. For cell viability assays with drug treatment, the cells were seeded onto 96-well plates and cultured overnight as described above. Subsequently, cells were treated with VT104 TEAD inhibitor (#HY-134956, MedChemExpress) at concentrations ranging from 10 to 10,000 nmol/L for 6 days.

### Migration assay

Cells were treated with PITX2 siRNA or control siRNA for 24 hours; then, 2 × 10^5^ cells/well were seeded onto the upper chamber of a 24-well Transwell insert (Corning) and incubated for 18 hours. The cells that migrated into the lower side of the filter were fixed and stained with DAPI (Vector Laboratories) and then counted under a fluorescence microscope.

### Scratch wound healing assay

Cells were seeded onto six-well plates at a density of 2 × 10^5^ cells/well and grown to confluence and then transfected with PITX2 siRNA or control siRNA overnight. Scratches were made with a 1 mL plastic pipette tip across the diameter of each well, and cells were cultured in serum-free conditions. After 24 hours, the cell-migrated areas in the scratch were quantified using ImageJ (RRID:SCR_003070).

### Sphere formation assay

Cells were seeded onto six-well ultralow attachment plates (Corning) at a density of 5,000 cells/well in Ham’s F12 containing 20 ng/mL EGF (#AF-100-15, PeproTech), 20 ng/mL basic fibroblast growth factor (#100-18B, PeproTech), and B-27 Supplement (#17504044, Invitrogen). Triplicate wells were prepared for each cell type. After 2 weeks, visible spheres were manually counted using a microscope.

### Clonogenic assay

Cells were plated onto 24-well plates at a density of 2,500 cells/well in triplicate and cultured at 37°C under 5% CO_2_ overnight. Subsequently, cells were treated with VT104 at 10 to 10,000 nmol/L concentrations or DMSO for 7 days and then fixed with 10% acetic acid in 100% methanol for 15 minutes. Fixed cells were stained with 0.5% crystal violet in 20% methanol for 1 hour. Stained colonies were washed with distilled water, dried, and then scanned using a document scanner. For quantification, crystal violet was dissolved in 10% acetic acid in water, and absorbance at 595 nm was measured in a plate reader.

### Histologic analyses and immunohistochemical staining

The protein expression levels of YAP and PITX2 in the primary EACSCC tissues were evaluated by immunohistochemistry (IHC). The antibodies used in this analysis were as follows: YAP (#WH0010413M1, Sigma Aldrich, RRID:AB_1844253) and PITX2 (#PA5-98817, Thermo Fisher Scientific, RRID:AB_2813430). The histology and the results of IHC were independently reviewed by three experienced pathologists (T. Hongo, T. Nakano, and K. Taguchi), and the expression scores for YAP and PITX2 were evaluated as previously described (14). Briefly, nuclear staining intensity was defined as “intensity score” (scored as 0–3), whereas the population of positive-staining cells was defined as “frequency score” (scored as 0–4). Samples with a YAP intensity score ≥2 were classified as the YAP-high group. PITX2 expression scores were defined by the following formula: (PITX2 intensity score) × (PITX2 frequency score), and samples with a score ≥6 were classified as the PITX2-high group. For IHC staining of mouse xenograft tumor tissues, formalin-fixed, paraffin-embedded specimens obtained from tumors were stained with PITX2 and Ki67 (#M7240, Agilent, RRID:AB_2142367), as previously described (31).

### Murine xenograft models

All animal procedures were performed in compliance with the Guidelines for the Care and Use of Experimental Animals established by the Committee for Animal Experimentation of Kyushu University. Five-week-old female BALB/cSlc-*nu/nu* mice were purchased from Japan SLC and maintained under specific pathogen-free conditions. A total of 1 × 10^6^ ONO2 cells expressing PITX2 or control cells were subcutaneously implanted in the flank. Tumor sizes were measured every 3 days using a vernier caliper and calculated using the following formula: tumor volume = (length × width^2^)/2. The mice were euthanized 14 days after implantation, and the collected tumors were fixed with 10% formalin.

### Statistical analysis

**Figure 1.**
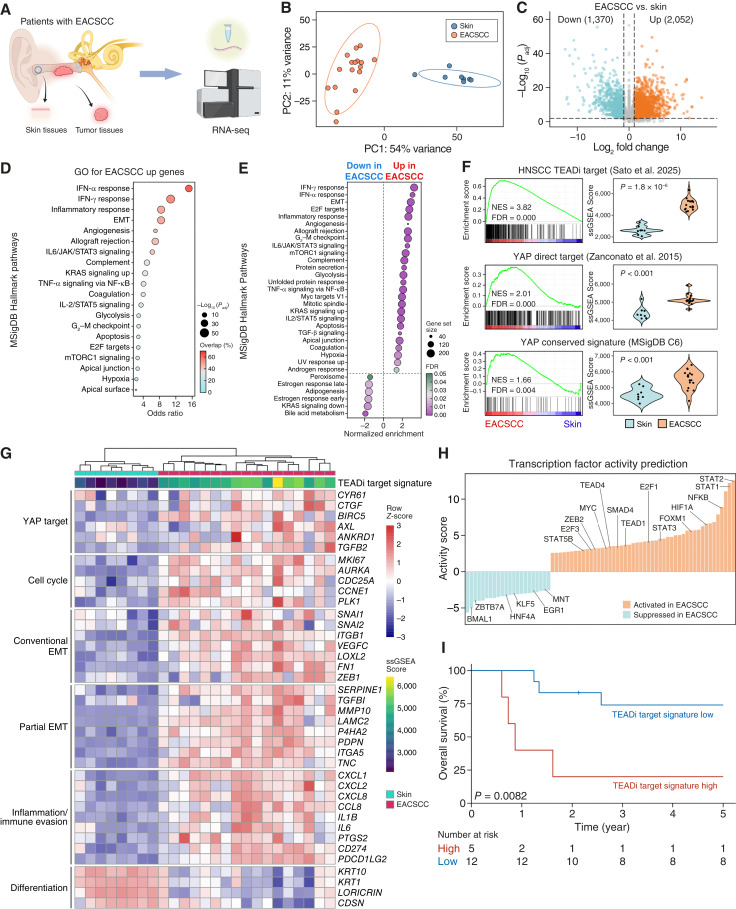
Transcriptomic analysis revealed hyperactivation of YAP signaling in EACSCC. **A,** Schematic representation of the experimental design of RNA-seq in EACSCC and noncancerous skin tissues. **B,** PCA for the RNA-seq data derived from EACSCC (*n* = 17) and noncancerous skin tissues (*n* = 8). **C,** Volcano plot representing DEGs between EACSCC and noncancerous skin tissues. **D,** GO analysis for the DEGs upregulated in EACSCC in the Molecular Signature Database (MSigDB) Hallmark pathways. **E,** GSEA representing gene sets positively or negatively enriched in EACSCC compared with noncancerous skin tissues in MSigDB Hallmark pathways. **F,** GSEA enrichment plots for the indicated YAP/TAZ-TEAD transcriptional target gene sets showing the enrichment between EACSCC and noncancerous skin tissues (left). Violin plots for ssGSEA scores for each group (right). **G,** The heatmap and hierarchical clustering for the selected genes in the indicated pathways. Gene expression levels were scaled among samples for each gene. The ssGSEA scores for the TEAD inhibitor target signature (Sato and colleagues; ref. 23) are shown at the top of the heatmap. **H,** Waterfall plots showing the predicted TF activities in EACSCC. **I,** Kaplan–Meier overall survival curves for the patients with EACSCC with TEAD inhibitor target signature high or low scores calculated by ssGSEA. PC, principal component. [**A,** Created in BioRender. Sato, K. (2026) https://BioRender.com/gwd1391.]

Statistical analyses were performed using R version 4.4.1. Overall survival and progression-free survival curves were plotted according to the Kaplan–Meier method and compared using the log-rank test. For survival analysis and the Kaplan–Meier plot in Fig. 1, YAP/TEAD activities were tested in Cox proportional hazard regression as a continuous variable, utilizing ssGSEA scores for the TEADi signature. Patients were then divided into “high” and “low” TEADi signature groups using *maximally selected rank statistics*, a statistical method that identifies the optimal cutoff point for a continuous variable with formal correction for multiple testing. This process was performed by the survminer R package (RRID:SCR_021094; refs. 23, 33). Multivariate analyses were performed using the Cox proportional hazards model to identify independent variables predictive of overall survival. For continuous variables, pairwise comparisons between groups were performed using a two-sided unpaired *t* test. Categorical variables were compared using Fisher’s exact test. The differences were considered significant when the *P* value was lower than 0.05. Data were represented as mean ± standard error of the mean (SEM).

## Results

### YAP/TEAD-driven transcriptional programs are hyperactivated in EACSCC

To clarify transcriptomic aberrations of EACSCC in an unbiased manner, we first performed RNA-seq utilizing RNA extracted from 17 treatment-naïve primary EACSCC and 8 noncancerous ear skin tissue specimens from 17 patients with EACSCC (Fig. 1A; Supplementary Table S1). PCA showed a clear separation between EACSCC and skin tissues, suggesting their distinct transcriptional profiles (Fig. 1B; Supplementary Fig. S1A). We detected 3,412 DEGs, including 2,052 upregulated and 1,370 downregulated genes in EACSCC (Fig. 1C; absolute value of fold change >2, FDR-adjusted *P* value < 0.01). GO analysis of upregulated DEGs showed significant enrichment of genes involved in inflammatory responses, epithelial–mesenchymal transition (EMT), and the cell cycle (Fig. 1D; Supplementary Fig. S1B). Similarly, GSEA indicated that pathways involved in inflammation, EMT, and cell-cycle progression are positively enriched in primary EACSCC (Fig. 1E). Strikingly, the previously established TEADi target signature in HNSCC (23), as well as YAP transcriptional target genes (24), were strongly enriched in EACSCC, suggesting the prominent role of YAP-driven transcriptional programs in EACSCC (Fig. 1F; Supplementary Fig. S2). The range of ssGSEA scores for the TEADi target signature observed in EACSCC was 4,327 to 6,321, whereas in skin, it was 2,068 to 3,337, similar to HPV-negative HNSCC in our previous analysis of The Cancer Genome Atlas RNA-seq data (23), which may suggest broad YAP/TEAD activation in squamous malignancies, including EACSCC. In addition to the upregulation of curated YAP/TEAD transcriptional target genes, conventional or partial EMT (p-EMT) associated genes (34), as well as immune-evasive chemokines/cytokines and immune checkpoint molecules (*CD274* and *PDCD1LG2*), were overexpressed, whereas differentiation-related genes were downregulated (Fig. 1G). Consistently, activated TFs in EACSCC included TEAD1 and TEAD4 (Fig. 1H). Of note, survival analysis of these 17 patients indicated that the TEADi target signature predicted poor survival of patients with EACSCC, suggesting that YAP/TEAD transcriptional activity is correlated with the malignant phenotypes of EACSCC (Fig. 1I). Together, transcriptome profiling suggested hyperactivation of the YAP/TEAD oncosignaling network in EACSCC.

### YAP-driven epigenetic aberrations in EACSCC

**Figure 2.**
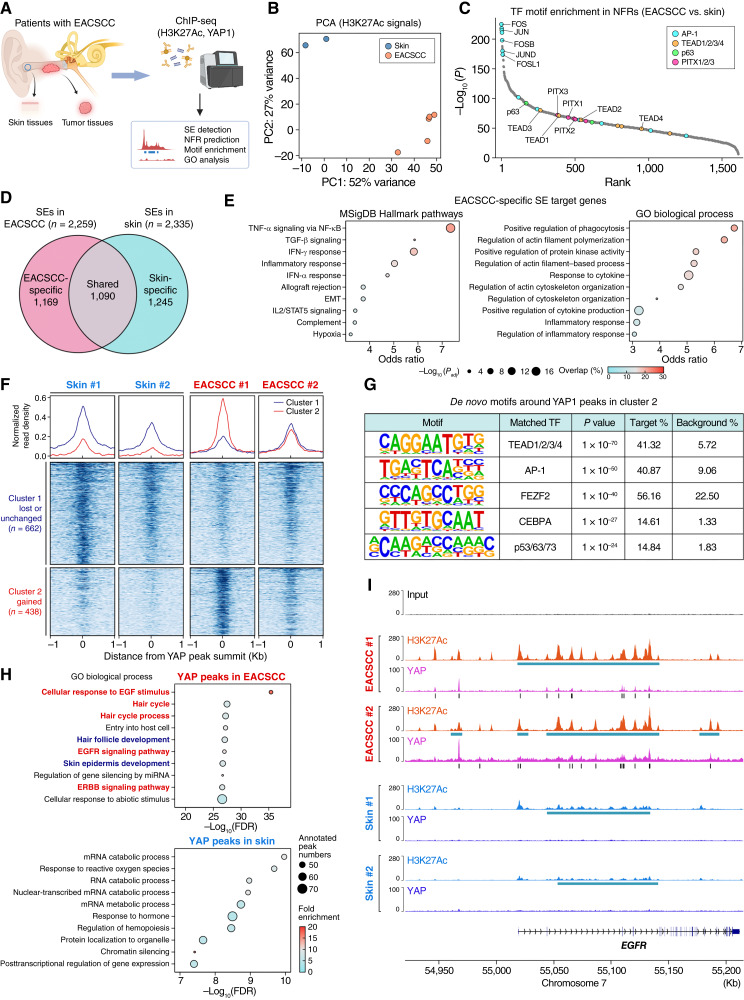
YAP-driven epigenetic reprogramming in EACSCC. **A,** Schematic representation of the experimental design of ChIP-seq in EACSCC and noncancerous skin tissues. **B,** PCA for the genome-wide H3K27Ac ChIP-seq signals derived from EACSCC (*n* = 5) and noncancerous skin tissues (*n* = 2). **C,** Enrichment of TF binding motifs in putative NFRs in EACSCC compared with noncancerous skin tissues. Selected TF motifs are highlighted. **D,** Venn diagrams for the SEs in EACSCC and noncancerous skin tissues. **E,** GO analysis for EACSCC-specific SE target genes in the Molecular Signature Database (MSigDB) Hallmark pathways (left) and GO biological process (right). **F,** Read density plots and heatmaps for YAP ChIP-seq signals in ±1 kb windows centered at YAP peak summits in EACSCC and noncancerous skin tissues. K-means clustering (*k* = 2) was performed to detect lost/unchanged (cluster 1, *n* = 662) and gained (cluster 2, *n* = 432) YAP signals in EACSCC. **G,***De novo* TF binding motif discovery analysis in 200 bp regions around YAP ChIP-seq peaks in cluster 2. **H,** GO analysis for the predicted transcriptional target genes of YAP in EACSCC and noncancerous skin tissues. Pathways involved in EGFR signaling and proliferation are highlighted in red, whereas pathways involved in differentiation are highlighted in blue. **I,** Genome browser snapshot representing H3K27Ac and YAP occupancy at the *EGFR* gene locus in EACSCC and noncancerous skin tissues. Detected SE regions and YAP peak summits are shown in blue and black bars, respectively. PC, principal component. [**A,** Created in BioRender. Sato, K. (2026) https://BioRender.com/t8rd8v4.]

Changes in chromatin accessibility and aberrant transcriptional machineries, including SEs, are one of the hallmarks of cancer (6, 35), and YAP is a potential regulator of this reprogramming (13, 36, 37). Considering this point, we performed ChIP-seq utilizing an antibody for H3K27Ac, a histone mark for active enhancers and promoters, as well as YAP in clinical EACSCC tissues and noncancerous ear skin tissues to clarify upstream mechanisms that regulate aberrant transcriptional programs in EACSCC (Fig. 2A). We successfully performed H3K27Ac ChIP-seq in five EACSCC samples and two noncancerous ear skin samples, detecting 53,400 peaks in EACSCC (range, 46,038–64,871), whereas there were 51,311 in skin (range, 42,967–59,655) on average. PCA of genome-wide H3K27Ac signal intensities showed a clear separation between EACSCC and skin, suggesting their distinct distribution of active transcription-regulatory elements (Fig. 2B). Based on these data, we predicted NFRs, accessible regulatory regions in enhancers and promoters, using a previously described bioinformatics algorithm (27). Motif enrichment analysis of NFRs showed significant enrichment of binding motifs for several TFs, such as the AP-1 family (e.g., JUN and FOS), p63, TEAD, and PITXs, which are reportedly involved in tissue regeneration and stemness (4, 38, 39) in EACSCC compared with skin, suggesting increased chromatin accessibility for these TF binding sites in this malignancy (Fig. 2C). We detected 1,024 and 1,021 SEs in EACSCC and skin on average, respectively (Supplementary Fig. S3A). We next calculated overlapping SE regions between samples and defined tumor-specific SEs, which are exclusively observed in EACSCC tissues, to explore their possible biological roles in EACSCC (see “Materials and Methods”; Fig. 2D). GO analysis for the putative transcriptional target genes of EACSCC-specific SEs indicated enrichment of oncogenic processes such as EMT, cell cycle, hypoxia, and inflammatory response, suggesting that upregulated genes in EACSCC tissues that we identified above (Fig. 1) could be regulated in part through the SEs (Fig. 2E). Subsequently, we sought to clarify YAP-driven transcriptional aberrations in EACSCC utilizing YAP ChIP-seq and integrating it with H3K27Ac ChIP-seq data. We were able to perform YAP ChIP-seq on two EACSCC tissues and paired two noncancerous skin tissues. Clustering analysis of YAP signals around YAP peaks indicated EACSCC-specific binding patterns of YAP (cluster 2; Fig. 2F). As expected, motif analysis for 200 bp regions around YAP peak summits in this cluster showed significant enrichment of DNA binding motifs for TEAD and AP-1, indicating that these TFs are predominantly forming complexes with YAP, as previously shown in cultured cells (Fig. 2G; refs. 24, 40). Notably, GO analysis of predicted YAP target genes showed strong enrichment of EGFR signaling, proliferation, and differentiation in EACSCC, which were not observed in skin (Fig. 2H). Unexpectedly, we observed significant YAP binding peaks with SE formation around the coding region for *EGFR* exclusively in EACSCC, suggesting that YAP/TEAD may directly regulate *EGFR* transcription through SEs (Fig. 2I). In addition, YAP binding peaks were observed in *EREG* (epiregulin) and *AREG* (amphiregulin), both of which are ligands for EGFR, as well as the EMT-promoting TF *SNAI2* and the immune-evasive chemokine *CXCL8* (Supplementary Fig. S3B–S3D). Overall, these results indicated that YAP could drive oncogenic transcriptional programs through SEs in EACSCC.

### PITX2, a potential alternative partner TF of YAP under TEAD-inhibited conditions, regulates oncogenic transcription in EACSCC

**Figure 3.**
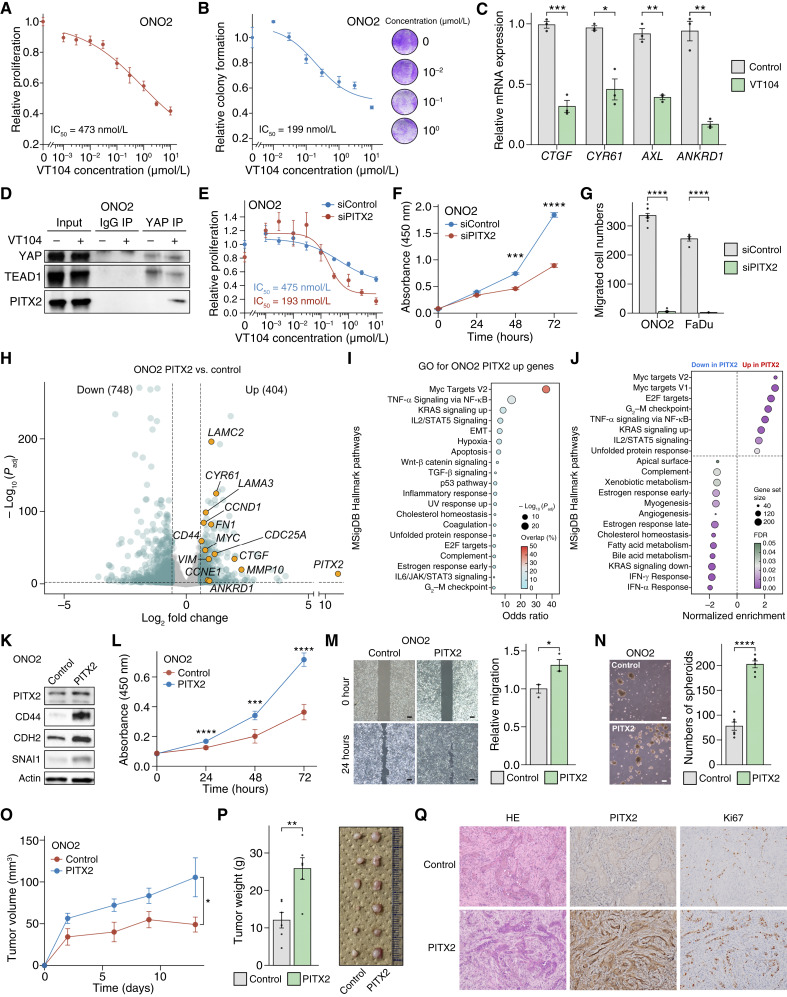
PITX2 acts as a potential partner TF of YAP in EACSCC. **A** and **B,** Dose–response curves of VT104 in ONO2 cells examined in a proliferation assay (*n* = 6; **A**) and a clonogenic assay (*n* = 3; **B**). **C,** qPCR for YAP/TAZ-TEAD target genes upon treatment with VT104 (1 μmol/L) for 24 hours in ONO2 cells *in vitro* (*n* = 3). **D,** YAP Co-IP experiment in ONO2 cells treated with VT104 (1 μmol/L) for 24 hours. **E,** Dose–response curves of VT104 in ONO2 cells upon siRNA-mediated knockdown against PITX2 (*n* = 6). The IC_50_ value for each group is shown. **F,** Proliferation assays in ONO2 cells upon siRNA-mediated knockdown against PITX2 (*n* = 3). **G,** Transwell migration assays in ONO2 and FaDu upon siRNA-mediated knockdown against PITX2 (*n* = 5). **H,** A volcano plot representing DEGs between PITX2-overexpressed and control ONO2 cells. Selected genes are highlighted as yellow dots. Genes with an adjusted *P* value < 0.01 and an absolute value of fold change >1.5 were considered significant. **I,** GO analysis for the DEGs upregulated in PITX2-overexpressed ONO2 cells compared with control cells in the Molecular Signature Database (MSigDB) Hallmark pathways. **J,** GSEA representing gene sets positively or negatively enriched in PITX2-overexpressed ONO2 cells compared with control cells in MSigDB Hallmark pathways. **K,** Western blots for the indicated proteins in PITX2-overexpressed and control ONO2 cells. **L,** Proliferation assays in PITX2-overexpressed and control ONO2 cells (*n* = 5). **M,** Wound healing assays in PITX2-overexpressed and control ONO2 cells (*n* = 3). **N,** Spheroid formation assays in PITX2-overexpressed and control ONO2 cells (*n* = 6). **O** and **P,** Growth curves for PITX2-overexpressed or control ONO2 xenograft tumors (**O**) and bar plots for tumor weight at endpoint (**P**; *n* = 6). The image of tumors is also shown. **Q,** Representative images for HE staining as well as immunohistochemical staining for PITX2 and Ki67 in the xenograft tumors. Data represent mean ± SEM. *, *P* < 0.05; **, *P* < 0.01; ***, *P* < 0.001; ****, *P* < 0.0001.

Our sequencing data provided a rationale for the hyperactivation of YAP in EACSCC and suggested that EACSCC could be dependent on YAP-driven transcriptional programs. Based on the results of YAP ChIP-seq in our clinical EACSCC samples, YAP predominantly interacts with TEAD, as previously reported *in vitro* and *in vivo* (41), suggesting that pharmacologic inhibition of the YAP–TEAD interaction may serve as a potential therapeutic approach for EACSCC. We sought to explore this question utilizing VT104, a small-molecule TEAD autopalmitoylation inhibitor, which blocks the YAP–TEAD interaction (42), in EACSCC-derived cells ONO2, previously established from primary EACSCC (31). As expected, VT104 inhibited the proliferation and clonogenicity of ONO2 (Fig. 3A and B), and several YAP/TEAD target genes were downregulated in qPCR upon VT104 treatment (Fig. 3C; Supplementary Table S2), indicating the dependencies on YAP/TEAD signaling in EACSCC cells. Consistent with our previous study of TEAD inhibitors in HNSCC (23), VT104 also suppressed the proliferation of representative HNSCC cells FaDu and SCC9, but HSC4 cells showed resistance to YAP/TEAD blockade (Supplementary Fig. S4A). Interestingly, TEADi target signature genes were significantly enriched in ONO2 compared with HSC4 and SCC9 in GSEA, and HSC4 indicated a relatively low TEADi target signature score compared with ONO2 and SCC9 (Supplementary Fig. S4B). These findings may suggest that basal YAP/TEAD transcriptional activity could be correlated with sensitivity to YAP/TEAD inhibition and that HSC4 might be less dependent on YAP/TEAD-driven transcriptional programs (31). However, whether alternative TFs could bind to YAP and be involved in oncogenic transcription under certain conditions is still controversial. Motif analysis in NFRs indicated that binding sites for several TFs are accessible in EACSCC but not in skin (Fig. 2C), suggesting the possibility that YAP may interact with TFs other than TEAD. One of the most significantly enriched TF binding motifs in accessible regions on enhancers included PITX2 TF, which is reportedly involved in heart muscle regeneration cooperatively with Yap1 in mice (38), as well as wound healing (4), oncogenic transcription, and stemness in cancer (39). Thus, we hypothesized that PITX2 could be an alternative partner TF of YAP and performed further analyses. Strikingly, YAP bound to PITX2 upon VT104 treatment, whereas dissociation between YAP and TEAD1 was observed in Co-IP assays (Fig. 3D). Binding between YAP and PITX2 was also observed in HSC4 HNSCC cells without TEAD inhibition, possibly suggesting a more predominant role of this TF in HNSCC and the presence of a YAP/PITX2 signaling axis (Supplementary Fig. S4C). Most importantly, siRNA-mediated knockdown of PITX2 significantly enhanced sensitivity to VT104 and reduced the proliferation of ONO2 cells compared with control (IC_50_: 475 nmol/L vs. 193 nmol/L; Fig. 3E). In addition, overexpression of PITX2 in ONO2 cells enhanced proliferation under VT104 treatment (Supplementary Fig. S4D). These data suggest that the YAP–PITX2 interaction may serve as a potential mechanism of resistance to pharmacologic YAP/TEAD blockade, and concurrent inhibition of YAP/TEAD and PITX2 could synergistically suppress EACSCC cell growth. Knockdown of PITX2 significantly reduced proliferation and clonogenicity, as well as migration in ONO2 and representative HNSCC cells FaDu, HSC4, and SCC9 (Fig. 3F and G; Supplementary Fig. S4E–S4G). To clarify the oncogenic function of PITX2 in EACSCC, we next performed RNA-seq in ONO2 cells stably overexpressing PITX2 and control cells. Notably, PITX2 induced the expression of genes involved in partial or conventional EMT (*VIM*, *LAMC2*, *LAMA3*, *MMP10*, and *FN1*), stemness (*CD44*), and cell-cycle progression (*MYC*, *CCNE1*, *CCND1*, and *CDC25A*). Unexpectedly, significantly upregulated genes included well-characterized YAP/TEAD target genes (*CYR61*, *CTGF*, and *ANKRD1*), suggesting that PITX2 may partially rescue YAP/TEAD-driven transcription (Fig. 3H; absolute value of fold change >1.5, FDR-adjusted *P* value < 0.01). GO analysis and GSEA indicated significant enrichment of gene sets involved in cell-cycle progression and EMT (Fig. 3I and J). Consistently, we observed upregulation of CD44, as well as EMT markers CDH2 and SNAI1 upon PITX2 overexpression by Western blotting (Fig. 3K). Of note, PITX2 overexpression significantly promoted proliferation, migration, and spheroid formation of ONO2 *in vitro* (Fig. 3L–N), as well as tumor growth *in vivo* (Fig. 3O–Q). These results suggested the oncogenic transcriptional ability of PITX2 in EACSCC.

### YAP and PITX2 predict poor prognosis of patients with EACSCC

**Figure 4.**
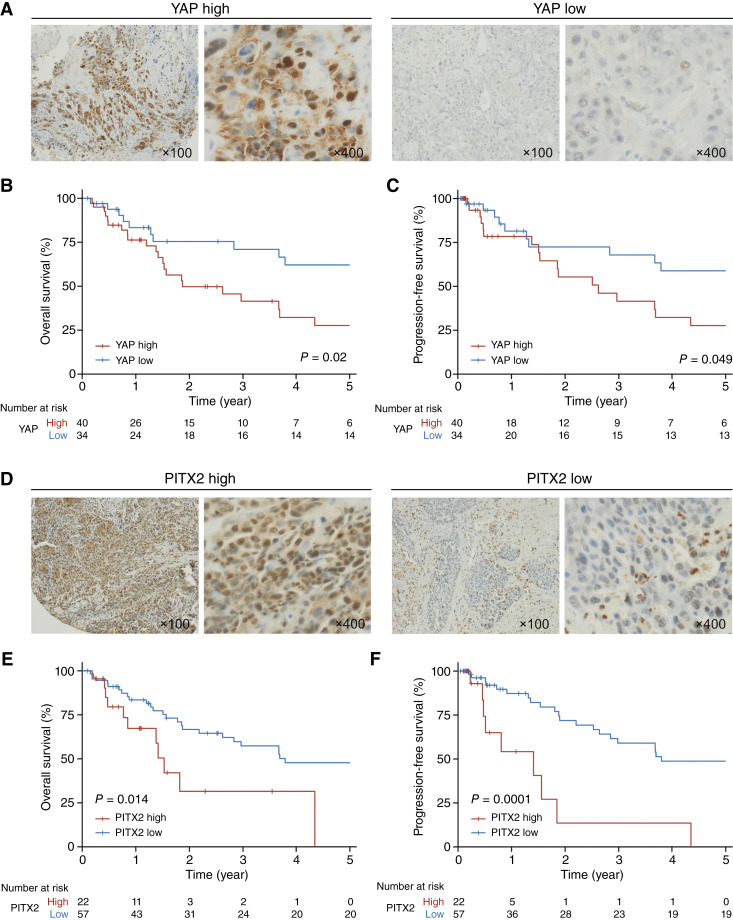
YAP and PITX2 predict poor prognosis of patients with EACSCC. **A,** Representative images of immunohistochemical staining for YAP in EACSCC tissues. **B** and **C,** Kaplan–Meier curves for overall survival (**B**) and progression-free survival of patients with EACSCC classified according to the YAP expression levels. **D,** Representative images of immunohistochemical staining for PITX2 in EACSCC tissues. **E** and **F,** Kaplan–Meier curves for overall survival (**B**) and progression-free survival of patients with EACSCC classified according to the PITX2 expression levels. *P* values were calculated using the log-rank test.

Finally, we examined the expression of YAP protein in treatment-naïve biopsy or surgically resected primary tumor tissue samples from 74 patients with EACSCC by IHC. Overexpression of YAP was observed in the nucleus of tumor cells in 40 out of 74 specimens examined (54%), suggesting that YAP is in its functionally active state (Fig. 4A). Patients with EACSCC with high expression levels of YAP showed significantly poorer overall and progression-free survival (Fig. 4B and C). We also evaluated PITX2 expression in EACSCC tissues (*n* = 79) and found that PITX2 was highly expressed in the nucleus of EACSCC cells in 22 out of 79 specimens (28%; Fig. 4D). Of note, high PITX2 expression was positively correlated with poor overall and progression-free survival, similar to the results of YAP IHC (Fig. 4E and F). In addition, YAP and PITX2 expression levels were positively correlated in the overlapping cases (*N* = 73; Fisher’s exact test, *P* = 0.022; Supplementary Fig. S5). Although statistical significance was not observed, both PITX2 and YAP were correlated with poor overall survival in multivariate analysis (*n* = 72; HR, 1.826; 95% CI, 0.976–3.416; *P* = 0.058 for YAP; HR, 2.200; 95% CI, 0.972–4.975; *P* = 0.059 for PITX2; Supplementary Table S3). These clinical data may support our sequencing and experimental findings, indicating that both YAP and PITX2 are prognostic factors for EACSCC.

## Discussion

The rarity of EACSCC has prevented the development of evidence-based therapeutic strategies as well as a comprehensive understanding of its biological and molecular backgrounds. One of the unique aspects of EACSCC is that this malignancy could be induced by chronic tissue damage and inflammation. This may be supported by our previous genomic study of EACSCC, in which tobacco- or UV-induced mutational signatures were not evident; instead, we identified predominant APOBEC signatures, which suggested a link to chronic inflammation (17), similar to aggressive cutaneous SCC in recessive dystrophic epidermolysis bullosa driven by an inflammatory microenvironment (43). Considering that aberrant and continuous hyperactivation of regenerative transcriptional programs in response to chronic tissue damage may fuel oncogenesis (6, 44), EACSCC could be addicted to this oncogenic transcription, and thus, this could be a therapeutic vulnerability that can be exploited for therapeutic purposes as well. However, unlike other types of malignancies in which transcriptomic and pathway-level alterations are well described in large-scale studies, the core transcriptional programs activated in EACSCC have not yet been clarified.

In this study, we provided evidence of hyperactivation of YAP-driven transcriptional programs in EACSCC. Indeed, RNA-seq analysis indicated that curated YAP/TEAD target gene sets were significantly enriched in EACSCC (23, 24), which was supported by ChIP-seq and IHC analyses of clinical samples. Notably, YAP/TEAD-driven transcriptional programs, as well as YAP nuclear expression, were correlated with poor prognosis in patients with EACSCC, suggesting that YAP hyperactivation may drive the malignant phenotype of EACSCC.

To date, no study has achieved YAP ChIP-seq in human clinical tissue samples. Our YAP ChIP-seq data may provide a better rationale for the hyperactivation of YAP/TEAD in human malignancies and suggest its downstream pathways, including EMT, chemokine production, and EGFR signaling. Specifically, SE formation and YAP binding in *EGFR*, as well as its ligands *EREG* and *AREG* loci, indicated that YAP/TEAD may directly control the transcription of these targets through SE. These findings may support the potential positive-feedback loop between YAP/TEAD and EGFR signaling, as recent studies have shown that YAP/TEAD promotes the transcription of the EGFR ligand *NRG1* (13) and that EGFR activates YAP/TEAD through the phosphorylation of a Hippo component (45). Moreover, H3K27Ac ChIP-seq indicated genome-wide SE formation and gained accessibility for oncogenic TF binding sites in EACSCC. Previous studies demonstrated that YAP recruits bromodomain-containing protein 4, an epigenetic cofactor essential for SE formation, to the enhancer and maintains an active chromatin state (36, 37, 46), suggesting that YAP could be a master regulator for SE-mediated oncogenic transcription. In this context, the co-occurrence of SE and YAP bindings, exclusively observed in EACSCC, may ndicate the crucial role of YAP-driven epigenetic reprogramming and transcriptional addiction to the Hippo-YAP/TEAD oncogenic signaling network in EACSCC.

Unexpectedly, we found that YAP bound to PITX2, a TF involved in stemness and tissue regeneration (38, 39), under pharmacologic YAP/TEAD blockade. Generally, TEAD is considered the most important YAP-binding TF, and the interacting mechanisms are well documented by a series of biological studies (9, 41), which ultimately resulted in the discovery and development of smTEADis (7, 23, 42, 47). However, the mechanistic consequence of YAP/TEAD inhibition is not yet fully uncovered, which may be context-dependent and cell type–specific (48). Our recent studies demonstrated that YAP/TEAD inhibition induced terminal differentiation of HNSCC and skin epithelial cells, which could be driven by other TFs such as KLF4, suggesting alternative transcriptional machineries other than YAP/TEAD (23, 49). Although the exact mechanism of YAP–PITX2 interaction still needs to be further elucidated, our findings suggest that PITX2 may rescue YAP/TEAD-driven oncogenic transcription, including proliferation, EMT, and stemness. Importantly, the function of PITX TFs has been mainly described in the context of tissue regeneration and wound healing (4, 38). Of interest, both PITX1 and PITX2 are expressed in oral mucosa and are involved in rapid wound healing, whereas these TFs are not expressed in normal skin, even in the regeneration process (4). In this regard, our experimental data, as well as the overexpression of YAP and PITX2 in EACSCC tissues, suggest a transcriptionally reprogrammed state of EACSCC and its unique oncogenic transcriptional networks, which may propose a potential therapeutic strategy cotargeting YAP/TEAD and YAP/PITX2 signaling axes.

Of note, IHC analysis of PITX2 demonstrated that nuclear expression of PITX2 protein significantly correlated with poor overall and progression-free survival, suggesting its contribution to aggressive phenotypes of EACSCC and potential as a prognostic biomarker for patients with EACSCC, which was further supported by our functional experiments on PITX2 in EACSCC and HNSCC cells. Interestingly, *PITX2* mRNA expression and hypomethylation of the *PITX2* promoter were reportedly correlated with poor prognosis for patients with HNSCC (50, 51). Similarly, PITX2 promoted stemness and proliferation of esophageal SCC (ESCC) cells and predicted poor prognosis for patients with ESCC (39), all of which may suggest its potential oncogenic role in squamous cancer.

In summary, we describe the transcriptomic and epigenetic aberrations of EACSCC and provide evidence that hyperactivated YAP mediates transcriptional programs that drive the malignant phenotypes in EACSCC. Our findings may contribute to a better understanding of YAP-driven epigenetic reprogramming not only in EACSCC but also in human squamous malignancies, as well as provide a mechanistic rationale for the future clinical development of novel therapeutic strategies, such as those targeting the Hippo-YAP/TEAD signaling network, for patients with EACSCC.

## Supplementary Material

Figure S1 Transcriptomic distances and gene ontology analysis in EACSCC and Skin tissues.

Figure S2 Single sample GSEA analysis in EACSCC and Skin tissues.

Figure S3 Super Enhancer formation and YAP bindings in EACSCC and Skin tissues.

Figure S4 The effect of TEAD inhibition and PITX2 knockdown in EACSCC and HNSCC cells in vitro.

Figure S5 Correlation between PITX2 and YAP expression in EACSCC tissues.

Table S1 Clinical data for EACSCC patients enrolled in RNA-seq analysis in Figure 1.

Table S2 Primer sequences for qPCR in Figure 3C.

Table S3 Multivariate analyses of YAP and PITX2 expression with clinicopathological factors for overall survival in the EACSCC patients in Figure 5.

## Data Availability

The RNA-seq datasets generated in this study have been deposited in the NCBI Gene Expression Omnibus with accession number GSE306447. The ChIP-seq datasets have been deposited in the Japanese Genotype-phenotype Archive with accession number JGAS000645. All other data are available in the main article or supplemental files, with the significant peak information in the ChIP-seq data available from the corresponding author upon reasonable request.
