# Supplementary material for: Aberrant Hippo-YAP/TEAD Signaling Drives Malignant Transcriptional Reprogramming in External Auditory Canal Squamous Cell Carcinoma
Source: Cancer Res Commun. 2026 Feb 2;6(2):260–72. doi: 10.1158/2767-9764.CRC-25-0626 (PMC12862246; doi:10.1158/2767-9764.CRC-25-0626)
Supplement: Figure S2 — Single sample GSEA analysis in EACSCC and Skin tissues. [file crc-25-0626_figure_s2_suppsf2.pdf]

# Figure S2

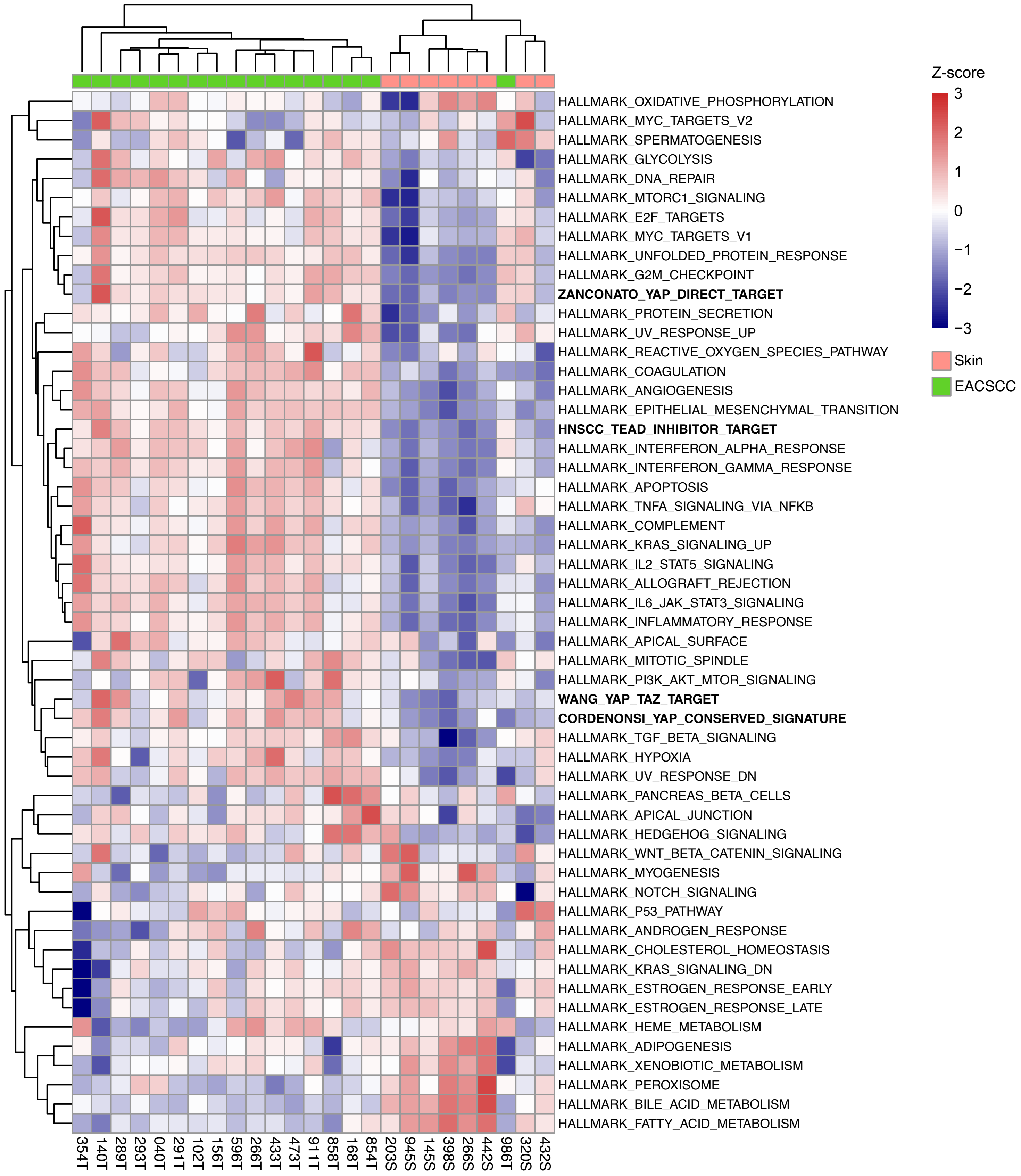


**Figure S2. Pathway-level transcriptomic aberrations of EACSCC.**
The heatmap for the scores from single sample Gene Set Enrichment Analysis in EACSCC and noncancerous ear skin tissues. Molecular Signature Database Hallmark genesets and YAP/TAZ-TEAD target genes were included for the analysis.
