# Supplementary material for: Aberrant Hippo-YAP/TEAD Signaling Drives Malignant Transcriptional Reprogramming in External Auditory Canal Squamous Cell Carcinoma
Source: Cancer Res Commun. 2026 Feb 2;6(2):260–72. doi: 10.1158/2767-9764.CRC-25-0626 (PMC12862246; doi:10.1158/2767-9764.CRC-25-0626)
Supplement: Figure S3 — Super Enhancer formation and YAP bindings in EACSCC and Skin tissues. [file crc-25-0626_figure_s3_suppsf3.pdf]

Figure S3

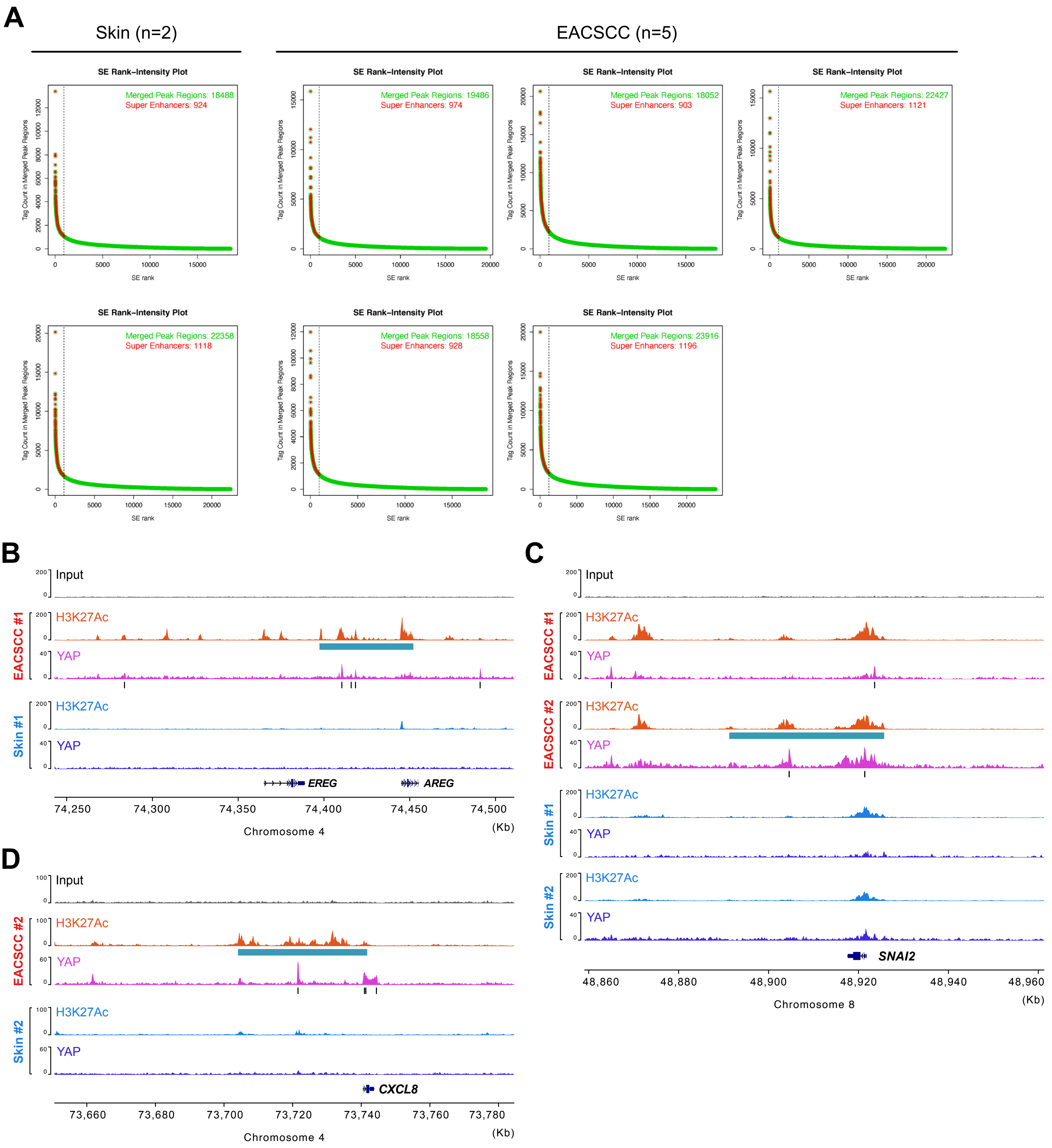

Figure S3. Super Enhancer (SE) formation and YAP binding in EACSCC.
(A) Enhancer regions (green) and SE regions (red) ranked by H3K27Ac signals at enhancers obtained by ROSE in noncancerous ear skin tissues (n=2) and EACSCC (n=5). **(B-D)** Genome browser snapshots for H3K27Ac and YAP occupancy at *EREG* and *AREG* (**B**), *SNAI2* (**C**) and *CXCL8* (**D**) coding regions. Blue and black bars represent SEs and YAP peaks, respectively.
