# Supplementary material for: Aberrant Hippo-YAP/TEAD Signaling Drives Malignant Transcriptional Reprogramming in External Auditory Canal Squamous Cell Carcinoma
Source: Cancer Res Commun. 2026 Feb 2;6(2):260–72. doi: 10.1158/2767-9764.CRC-25-0626 (PMC12862246; doi:10.1158/2767-9764.CRC-25-0626)
Supplement: Figure S4 — The effect of TEAD inhibition and PITX2 knockdown in EACSCC and HNSCC cells in vitro. [file crc-25-0626_figure_s4_suppsf4.pdf]

Figure S4

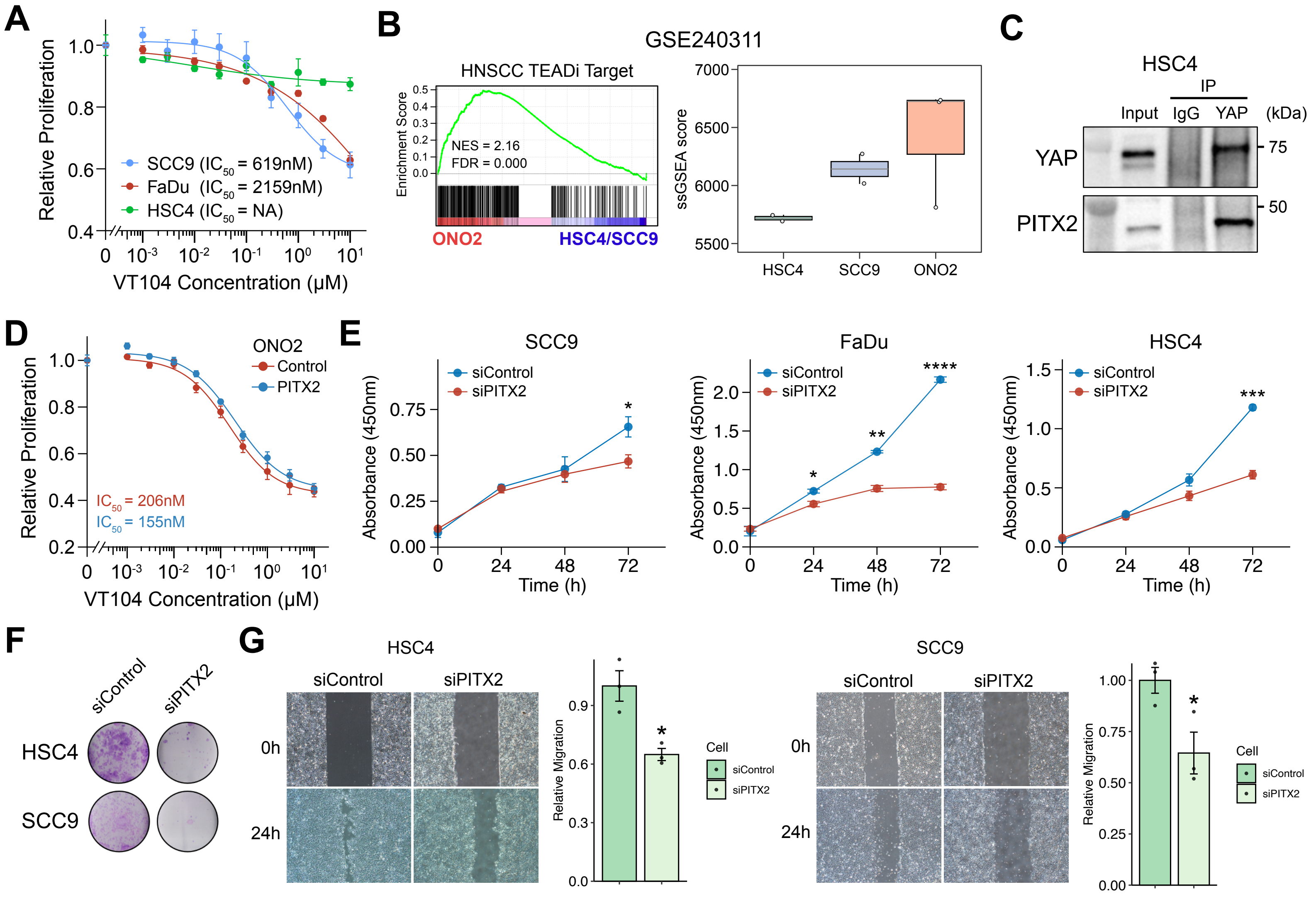

Figure S4. Knockdown of PITX2 suppresses proliferation and migration of SCC cells *in vitro*.
(A) Dose-response curves of VT104 in the indicated HNSCC cells examined in proliferation assay. **(B)** GSEA plot comparing ONO2 with HNSCC cells (left) and boxplots for ssGSEA scores for TEADi target signature for HSC4 (n=2), SCC9 (n=2) and ONO2 (n=3) calculated from RNA-seq data of our previous study (Ref.31, GSE240311). **(C)** Co-immunoprecipitation utilizing YAP antibody in HSC4 HNSCC cells. Western blot for YAP and PITX2 are shown. **(D)** Dose-response curves of VT104 in PITX2-overexpressing and control ONO2 cells examined in proliferation assay. **(E)** Proliferation assays in the indicated HNSCC cells treated with siPITX2 and control siRNA. n=3 for each group. **(F)** Clonogenic assays in HSC4 and SCC9 cells treated with siPITX2 and control siRNA. **(G)** Migration assays in HSC4 and SCC9 cells treated with siPITX2 and control siRNA. n=3 for each group. *$P < 0.05$, **$P < 0.01$, ***$P < 0.001$, ****$P < 0.0001$.
