# Supplementary material for: Aberrant Hippo-YAP/TEAD Signaling Drives Malignant Transcriptional Reprogramming in External Auditory Canal Squamous Cell Carcinoma
Source: Cancer Res Commun. 2026 Feb 2;6(2):260–72. doi: 10.1158/2767-9764.CRC-25-0626 (PMC12862246; doi:10.1158/2767-9764.CRC-25-0626)
Supplement: Figure S5 — Correlation between PITX2 and YAP expression in EACSCC tissues. [file crc-25-0626_figure_s5_suppsf5.pdf]

Figure S5

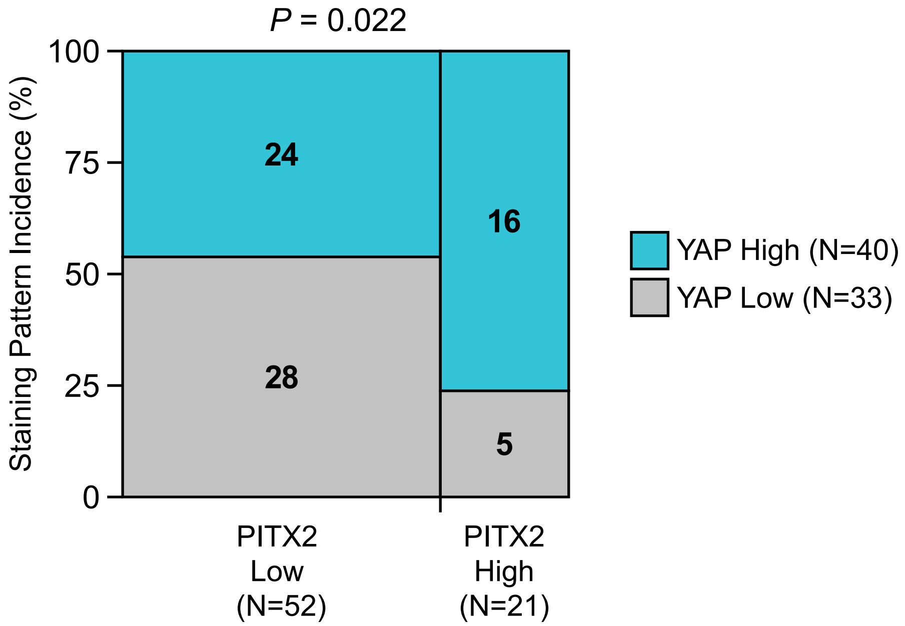

Figure S5. YAP and PITX2 expression levels are positively correlated in EACSCC tissues. Mosaic plot summarizing YAP and PITX2 expression in EACSCC tissues for the indicated number of EACSCC patients (N = 73). The p-value for the association between the parameters was calculated via Fisher's exact test.
