# Supplementary material for: Aberrant Hippo-YAP/TEAD Signaling Drives Malignant Transcriptional Reprogramming in External Auditory Canal Squamous Cell Carcinoma
Source: Cancer Res Commun. 2026 Feb 2;6(2):260–72. doi: 10.1158/2767-9764.CRC-25-0626 (PMC12862246; doi:10.1158/2767-9764.CRC-25-0626)
Supplement: Table S1 — Clinical data for EACSCC patients enrolled in RNA-seq analysis in Figure 1. [file crc-25-0626_table_s1_suppst1.docx]

**Table S1. Clinical Characteristics of EACSCC Patients enrolled in RNA-seq analysis.**

| Patient ID | Gender | Age | Primary site | T | N | M |
| --- | --- | --- | --- | --- | --- | --- |
| 168T | M | 72 | L | 1 | 0 | 0 |
| 102T | M | 66 | L | 2 | 0 | 0 |
| 293T | M | 71 | L | 2 | 0 | 0 |
| 354T | F | 78 | R | 3 | 0 | 0 |
| 040T | F | 65 | R | 3 | 0 | 0 |
| 854T | M | 83 | R | 3 | 0 | 0 |
| 858T | F | 66 | R | 3 | 0 | 0 |
| 596T | F | 55 | R | 4 | 0 | 0 |
| 291T | M | 59 | R | 4 | 0 | 0 |
| 433T | F | 48 | L | 4 | 0 | 0 |
| 156T | F | 60 | R | 4 | 0 | 0 |
| 986T | F | 66 | R | 4 | 0 | 0 |
| 289T | F | 75 | L | 4 | 1 | 0 |
| 473T | M | 56 | R | 4 | 0 | 0 |
| 911T | F | 33 | R | 4 | 0 | 0 |
| 266T | M | 66 | R | 3 | 0 | 0 |
| 140T | F | 67 | R | 4 | 2b | 0 |

M, Male; F, Female; L, Left; R, Right.
