# Supplementary material for: Aberrant Hippo-YAP/TEAD Signaling Drives Malignant Transcriptional Reprogramming in External Auditory Canal Squamous Cell Carcinoma
Source: Cancer Res Commun. 2026 Feb 2;6(2):260–72. doi: 10.1158/2767-9764.CRC-25-0626 (PMC12862246; doi:10.1158/2767-9764.CRC-25-0626)
Supplement: Table S2 — Primer sequences for qPCR in Figure 3C. [file crc-25-0626_table_s2_suppst2.docx]

**Table S2. Primers used for qPCR in this study.**

| Target gene | Forward primer (5’-3’) | Reverse primer (5’-3’) |
| --- | --- | --- |
| *CTGF* | TGTGTGACGAGCCCAAGGA | TCTGGGCCAAACGTGTCTTC |
| *CYR61* | CAGGACTGTGAAGATGCGGT | GCCTGTAGAAGGGAAACGCT |
| *AXL* | AGCACACGCGTAAACAACAC | GTTATGGGCTTCGCAGGAGA |
| *ANKRD1* | CGCCCGAGATAAGTTGCTCA | GGTTCAGTCTCACCGCATCA |
| *RPS18* | AGTCCCTGCCCTTTGTACACA | CGATCCGAGGGCCTCACTA |
