# Supplementary material for: Aberrant Hippo-YAP/TEAD Signaling Drives Malignant Transcriptional Reprogramming in External Auditory Canal Squamous Cell Carcinoma
Source: Cancer Res Commun. 2026 Feb 2;6(2):260–72. doi: 10.1158/2767-9764.CRC-25-0626 (PMC12862246; doi:10.1158/2767-9764.CRC-25-0626)
Supplement: Table S3 — Multivariate analyses of YAP and PITX2 expression with clinicopathological factors for overall survival in the EACSCC patients in Figure 5. [file crc-25-0626_table_s3_suppst3.docx]

**Table S3. Multivariate analyses of YAP/ PITX2 expression and clinicopathological factors affecting 5-years overall survival in EACSCC cohort (N=72).**

| Factor | HR (95%CI) | P-value |
| --- | --- | --- |
| Age (≥65/<65) | 1.521 (0.830-2.788) | 0.174 |
| Gender (Male/Female) | 1.273 (0.624-2.598) | 0.506 |
| Histology (≤moderate/≥well to moderate) | 0.687 (0.316-1.494) | 0.344 |
| T stage (T4/T1-3) | 0.712 (0.394-1.287) | 0.261 |
| Lymph node metastasis (Present/Absent) | 2.381 (1.042-5.443) | 0.039* |
| Distant metastasis (Present/Absent) | 5.346 (0.6118-46.723) | 0.129 |
| YAP expression (High/Low) | 1.826 (0.976-3.416) | 0.058 |
| PITX2 expression (High/Low) | 2.200 (0.972-4.975) | 0.059 |

*Statistically significant.
